# Three Years with the Army of the Potomac—A Personal Military History

**Published:** 1912-03

**Authors:** William Warren Potter

**Affiliations:** Buffalo, N. Y., Brevet Lieutenant Colonel, U. S. Volunteers; Surgeon in Charge of First Division Field Hospital Second Army Corps; Surgeon 57th Regiment New York Volunteers; Assistant Surgeon 49th Regiment New York Volunteers; Recorder Second Division Hospital Sixth Army Corps, etc., etc.


### Three Years with the Army of the Potomac—A Personal Military History

BY WILLIAM WARREN POTTER, M.D., BUFFALO, N. Y., BREVET LIEUTENANT COLONEL, U. S. VOLUNTEERS; SURGEON IN CHARGE OF FIRST DIVISION FIELD HOSPITAL SECOND ARMY CORPS; SURGEON 57TH REGIMENT NEW YORK VOLUNTEERS; ASSISTANT SURGEON 49TH REGIMENT NEW YORK VOLUNTEERS; RECORDER SECOND DIVISION HOSPITAL SIXTH ARMY CORPS, ETC., ETC.

July 1. This morning we were served with orders to be ready to move at a moment's notice, and stood to arms waiting the final order to march until afternoon. Meanwhile, we could see the bursting of the shells above the tree-tops beyond Gettysburg, which proved the beginning of the great battle. While here General Hancock was directed to turn the command of the 3d Corps over to General Gibbon, and proceed post-haste to Taneytown to report to the Commander-in-Chief. The troops finally received orders to proceed thither also, where we arrived about dark. Here we learned that, owing to the death of General Reynolds of the 1st Corps who had been killed that day, General Hancock had been sent to Gettysburg to assume command of all the troops then present on the field. We little understood the full import of this important assignment at the time, but it proved a duty of vast moment for Hancock, as well as a fortunate selection for the Army of the Potomac. It appears that Hancock soon brought order out of the chaos which the death of Reynolds had precipitated, and decided it a proper position for the Army of the Potomac to take up as a defensive line. He so advised Meade, who at once ordered up the whole Army. After resting for an hour at Taneytown we proceeded on our way to Gettysburg, following after our restive and famous Commander as fast as our legs could carry us. We halted for a rest between eleven and twelve o'clock, and I sat down upon a haycock in a field along the roadside, to rest and graze my horse. General Zook soon joined us, sitting on the hay beside me, and we entered into conversation about the importance of the work before us. The moon would occasionally break through the flying clouds,

"The air was refreshing,
"And the ripe harvest of the new-mown hay
"Gave it a sweet and wholesome odor."

The General had bought a horse in Maryland that day, for which he paid $250.00. It was a handsome animal, and he said it was a good purchase if he lived through the impending battle; but if he should be killed, in that event, he desired the horse sold. I inferred that he felt apprehensive for his own welfare, and that he might not survive; so I cheered him with a few encouraging words, reminding him of his previous good fortune in battle, etc. Alas! in less than a day he had received his mortal hurt. We soon started on our weary way, and a little after daylight in a misty rain marched upon the field of Gettysburg, taking position on the left-center of line. Just after our regiment had assumed its appointed position, I received an order to report to the Division Hospital as Assistant to Dr. C. S. Wood, one of the chief operating surgeons of the Division. The place selected for the hospital was in an oak opening east of the Taneytown Road, between it and the Baltimore Pike; and, after breakfast, I laid down and slept for several hours, as all was quiet.

This was Thursday, July 2. I awoke about noon, and soon we had dinner; but still there was no fighting. Finally, taking advantage of the dullness and quietude, I mounted my horse and rode over to General Mead's headquarters at the cottage on the Taneytown Road. I hitched to the palings of the fence and went inside the yard, where I met several officers of my acquaintance, visiting with them for perhaps an hour. I expressed a desire to take a look at the field, and was advised to proceed to Cemetery Hill, three quarters of a mile north, where I could get the best view. I did so, and soon became absorbed in the wonderful scene before my eyes, which I surveyed with the aid of a field glass. Looking directly west into the enemy's lines, I saw, in a gap in the woods, troops moving towards our left in considerable numbers. I could readily distinguish the regiments as they passed, and felt sure at the time that the movement was of serious import. Sweeping my glass still farther to the left, I soon saw a line of battle advancing towards the front, which proved to be the 3d Corps moving to take possession of the Emmetsburg Pike. It had been cloudy all day until now, but the sun just then burst through the clouds and glinted across the long line of bayonets; while the colors, unfurling and waving in the bright sunlight, presented a most entrancing sight to the observer. But in a moment this beauty of the parade was changed to the mad clamor of actual warfare; for the enemy's batteries opened as soon as the line had advanced sufficiently far to give them enfilading range. The first smoke brought forth the exclamation from a voice near me, "There, General, go the enemy's batteries," and on looking around to my left and rear, I saw Generals Doubleday and Howard in conversation together, and it was Doubleday who had spoken.

I soon became satisfied myself that they were the enemy's batteries we saw, for the shells commenced coming towards Cemetery Ridge and Hill; so I turned my horse into the road and started on my way back to the hospital. I halted again at Meade's headquarters on the way back, and went into the yard for a moment; but soon everybody there began to take to horse, Meade for the front, Pleasanton for his cavalry on the left, and other officers for their various posts and duty. The fact is, the fire fairly drove them out. I followed Pleasanton who was going in the direction of the hospital, and on the way the shells burst over and around us in frightful rapidity and proximity; but no one was hurt as far as I could observe. Arriving at the place where I had left the hospital, not a person was to be found, and no uaces of it could be discovered. I began searching for it, and soon found its new location near a stream protected by a ridge from great danger of shells, though we were somewhat nearer the lines than before. It seems that, during my absence, the hospital was literally shelled out of its first position, and this new place was sought out as a necessity.

The advancing line which I had just seen proved, as I remarked a little way back, to be General Sickles' Third Corps moving to take up position on the Emmetsburg Road. The enemy, too, had been manoeuvering for an attack upon our left all the afternoon; so these two mighty forces came together with an impact, which made the woods resound with the crash. Sickles soon became nearly overpowered, and Hancock went to his rescue with a part of the 2d Corps. The first Division caught the heaviest of the blow, many killed and wounded were the result, and the latter were now being brought to the hospital in great numbers. While busily engaged in their care, I received word that General Zook had been grievously wounded, and, was being moved to a farm house on the Baltimore Pike. I immediately repaired to the place and discovered at once that he was fatally shot, a shell having torn open his left shoulder and chest, exposing the heart-beats to observation. I remained with the Genenral until about eleven o'clock P. M., and left word with Lieutenant Favill, of Zook's staff, that I would come again in the morning should be survive the night, which now looked very improbable. Soon after leaving the house I met a detail of forty soldiers bearing General Sickles, whose thigh had been amputated on the field that evening, to Westminster, twenty-five miles away. This expedient no doubt saved his life, as to have been driven thither in an ambulance would probably have proven fatal.

After working till the small hours of the morning of the 3d, I laid down and obtained a little sleep, but was up and at it again by daylight. After assisting at many operations, as well as making a number myself to spell the chief operator, I once more repaired to General Zook's side finding him still alive, but rapidly sinking. He lingered, however, until near two o'clock P. M., passing away without a murmur of complaint, and in the full possession of all his mental activity. The famous cannonade, the prelude to Pickett's heroic charge, was in full blast at this hour, and Zook breathed out his life amidst the uproar and mad clamor of two hundred guns—the grandest combat of field artillery the world ever beheld, or held its breath to listen to. As soon as the General breathed his last I made haste for the front, where I knew my services were needed! and I arrived at the hospital not a moment too soon. After the sound of the cannon died away there was a short interval of comparative silence; then came the infantry battle. The 2d Corps received the weight of the shock, and our hospital was soon overflowing with wounded men, in the care of whom we were busy all night. I took a short nap near morning, but began work again soon after daylight the 4th. A heavy rain poured in upon us during the night, continuing during the 4th, and nearly flooding us out. During the 4th, in the midst of a heavy shower, I amputated the thigh of a rebel captain whose name I have forgotten. He belonged to a North Carolina regiment, was full of hope and courage, and I have no doubt he recovered. So the work went on for the next three days until, on the 7th, we were relieved by surgeons from Washington and the North.

### THE RETREAT AND PURSUIT OF LEE.

The Army was moving to intercept Lee, if possible, and we must go with the troops, where our services were likely to be needed at any time. General Meade did not permit our hospital wagons to come up until the battle was over, so we were not able to shelter our wounded very well, but in all other respects they were well cared for.

July 9. Yesterday it rained nearly all day, and quite hard for a considerable part thereof; nevertheless, we made a march of twenty-five miles, reaching Frederick about four P. M. Captain James D. Brady, of the staff, and I visited the city in the evening, but all was confusion in the town and we returned to camp, tired and disgusted with our attempt at recreation. Today we are destined for Crampton's Pass, where the Sixth Corps had a battle on the 14th of September last. I stopped at a farm house near noon, owned by a man named Roderick, and wrote a letter home. The family were very kind to me, inviting me to visit them after the war, which I partly promised to do.

July 12. We are again positioned before the enemy near Jones's Cross Roads, having come through a pass in the South Mountain, and are now about five miles from Williamsport near the road leading from Sharpsburg to Hagerstown. Our skirmishers are "at work" about half-a-mile to our front, the nature of the fire indicating that they are meeting quite stubborn resistance.

The 13th. We were busy all day perfecting our breastworks and otherwise preparing for active work with the enemy, and at night received orders looking to an attack upon his position at dawn next morning; but when we awoke on the 14th we found the enemy gone.

July 14. We followed on the heels of the retreating foe as fast as foot, hoof, and wheel would carry us, the 57th being deployed by skirmishers; and before night the First Division had captured nearly 2,000 stragglers from their rear guard. The recent rains made the roads and fields one vast sea of mud, and when we bivouacked at night we were a sight to behold. Every officer and man was covered with mud from his heels to his head, and the animals were as though they had been plastered.

July 16. In the field near Harper's Ferry. We bivouacked on the Virginia side of the Potomac near Williamsport Tuesday night, the 14th, and moved down the river to this point yesterday. We returned to the Maryland side of the river, and passed through Sharpsburg on our way hither, where we saw evidences of the battle of last September, in the yet unrepaired damage to the church and some of the houses. We are in that delightful spot known as "Pleasant Valley," and need rest from the fatigues of the hard fighting, and the long and rapid marching, day and night, of the past month; but I don't think we will be allowed to remain here very long.

July 17. It looks as though we would push into Virginia again tomorrow although we have been quiet today. We left Pleasant Valley Saturday morning, July 18, and marched every day until the 24th, save one, which brought us to Manassas Gap. On the 24 we rested for a few hours in the Gap the enemy being not far away, and ammunition was issued to the troops at two o'clock P. M., Dr. Corry, my second Assistant Surgeon, left today for Washington on sick leave and I forwarded a letter by him to be sent home.

Monday, July 27. Near Warrentown Junction. We have made long and tedious marches for the past ten days, the heat being very oppressive, but we are fortunately resting today. On the night of the 25th we camp at White Plains, where the 6th Corps stopped for three days last fall. Yesterday we marched twenty miles in the broiling sun, passing Warrentown about five P. M., and reaching this camp at ten o'clock at night. Warrentown is the prettiest Southern village I have seen, is delightfully located, and possesses some fine buildings. A great many of our men are barefooted, and some are half naked; but we may get supplied here. Our horses have been several days without forage, subsisting entirely upon grass; and as for myself, blackberries, of which there is an abundant supply, have been my chief article of diet for a week. We have had no mail since crossing the Potomac, but Lieutenant Snyder, Quartermaster of the 57th, goes to Washington tomorrow to procure clothing for the Division, and I can send a letter by him to keep them at home advised of the fact that I am still alive and well.

July 28. We have received two mails yesterday, with home dates to the 22. My wife writes as though she was anxious for me to leave the service; but the recent successes that have attended our Arms, both east and west, have inspired me with new courage. I have always thought the Army of the Potomac would strike the final blow at the Rebellion, and I have a strong desire to be "in at the death"; so I shall stick. Thus far I have been present at every battle this Army has fought since its organization under McClellan, and I take some pride in maintaining such a good record. No doubt, many find fault because Lee's army was not destroyed before we recrossed the Potomac, but when the entire facts are known, General Meade will be fully justified in the course he pursued. It is useless for me to endeavor to vindicate the Army of the Potomac, or its commanders: history will do us full justice, I have no doubt.

July 30. Still near Warrentown Junction. Yesterday, in company with Colonel Chapman, I paid a visit to the 11th Corps which is near us, where I saw Gad Parker, John McCray and others. Parker tells me that John Boyd, who belongs to his Company, was severely wounded at Gettysburg. We also called upon Lieutenant Colonel Stewart L. Woodford, of the 127th, New York, who is a friend of Colonel Chapman. The weather is cooler as we have had rain, and we are quiet; but I think we shall move again within a day or two.

August 1. We are again within six or seven miles of the Rappahannock at a place called Morrisville, having moved from near Warrentown Junction day before yesterday. We are opposite Kelley's Ford where we may cross, but are now simply guarding the passage. The weather is extremely hot, but I am in good health and spirits. Captain James C. Bronson left for New York today, to fetch on the drafted men for our command. How I envey his prospects of numerous juleps, ices, etc.

August 3. Morrisville was formerly a village of two houses, but one having been totally destroyed by fire, the "town" is only half its original size. I took a ride in company with Quartermaster Snyder to Elkton yesterday, four miles distant towards Warrentown Junction, going through a region infested by guerillas; but we returned in safety at seven-thirty P. M., not having seen one of that muchdreaded "chivalrous gentry." Lieutenant E. L. Palmer, of the 57th, in charge of a picket detail, disappeared on the same road we travelled, night before last, about half way between here and Elkton and is supposed to have been captured by the guerillas. Sickness is increasing, owing to the heat; today I report six men on the sick-list, more than at any time since we left Falmouth. Major Bull, 66th New York, had an adventure with the guerillas near Warrentown, when we were approaching that place a few days ago, an account of which appears in the New York Herald. (It will be found in my scrap-book.) My Brigade was in the rear of the wagon train that day guarding it, and the 57th was the rear regiment. I was in conversation with Major Bull near the broken-down ambulance spoken of not five minutes previous to the appearance of the guerillas, and came very near being mixed up in the affair myself. Our tents are now pitched in the edge of a beautiful wood, and we are delightfully situated in every way, but no telling how long we shall remain so.

August 6. Day before yesterday we changed our camp, moving about a mile and a half, for the purpose of getting better ground, and having water more convenient. Our Regimental Headquarters are now delightfully located on a beautiful knoll, upon the green summit of which stands a large cherry tree, and our tents are pitched around the tree facing inwards, which affords us shade all day long. I hope and believe we shall not have any fighting for a month, which, if my predictions fall true, will give us a much needed rest this hot weather.

APPOINTMENT TO THE CHARGE OF THE HOSPITAL.

August 8. Today, while we were sitting around our cherry tree, enjoying our post-prandial dish of conversation, an orderly rode up and placed in Colonel Chapman's hands an order, which, upon opening, proved to be an appointment for me, placing me in charge of the field hospital of the First Division, relieving in that duty Surgeon George L. Potter, of the 145th Pennsylvania Volunteers. I went to Headquarters immediately to learn the particulars of the change, and to arrange for the transfer of the property, which will take place tomorrow when I assume charge. My tent leaked badly last night, the water saturating my bed in places; but I shall have the tent condemned and get another. We are now living on green corn, cucumbers, fresh bread, canned tomatoes, peaches, etc.; so we need not complain.

August 10. I am very busy now putting the hospital in order, and systematizing the work. The place I hold is one of hard work and responsibility, but I do not complain; many an older man than I might feel proud to get the position, and I shall endeavor to discharge the duties pertaining to it in such a manner as to merit, and I hope receive, the commendations of my superior officers.

August 12. I regret to hear that Frank Folsom is drafted, but he will, no doubt, get relieved from serving on account of his health. I have thirty men on fatigue duty, building cots, ditching, building arbors across the front of the hospital, and otherwise making it comfortable.

August 14. Today has been cooler, with a shower just at night. I harnessed my horse and hitched him before a top buggy, that was captured by the Provost Marshall, and drove him out four miles and back today. It was considered quite an event, many looking on with interest and enjoying the unusual scene. The papers report the 6th Corps at Warrenton, as having a great time giving balls and parties, which the women of that village attend. It is reported in the Buffalo papers received tonight, that Lyman Bass and Oscar Folsom have both been drafted. They will, of course, both escape service by some device or other. It is also rumored that General Hancock will be appointed to the command of the Army of the Potomac as soon as he is able to take the field again. I hope this may prove true, but I question very much whether the Government will make another change in commanders soon, unless there should arise strong reasons therefor.

August 16. Colonel Chapman has received a letter from Lieutenant Palmer, who was captured on picket two or three weeks ago. He is now in Libby Prison, Richmond. We have rumors of a speedy move, but I have received no orders in regard to the hospital yet, though the troops are under orders to be ready to move at a moment's notice, I hear.

August 18. A cool, comfortable day comparatively. I have bought General Zook's saddle cloth which, with a new russet bridle obtained in Washington, sets off my black horse well, and makes him look quite handsome. I get my pay every month now, which is one of the advantages of being detached from the regiment on this kind of service.

August 20. I went down to Bealton Station today, and sent my July pay home by express. While there I fell in with an artist in tintypes, and so had some pictures of my horse taken.

These will be found among my collection of war pictures. On the way back to camp Lieutenant Mitchell of the Ambulance Corps, who was with me, found a pocket-book containing $75.00 or $80.00 in greenbacks. He sought, but never found, an owner for it. Calling at one of the houses in our neighborhod a few days ago, one of the women of the household gave me a sample of cloth which she wove, and like which was the dress that she was wearing at the time. It is called "Dixie Gingham," and I sent the sample home.

August 21. Yesterday I witnessed the execution of a deserter in the 2d Division—the first time the death sentence has been invoked in the 2d Corps. It was a melancholy spectacle, but a just way to deal with such dangerous men. We are having quiet times now. The hospital is running smoothly, and the entire command is in very good health. We are beginning to receive some of the fruits of the draft now going on in the North, which will reinforce our thinned and weakened ranks. A motley group of these raw soldiers arrives almost daily, a goodly number which seek the hospital upon the first excuse, and some of them needfully, as they become ill from the change of climate, as well as a radical revolution in their mode of living. The old soldiers are inclined to look with a jealous eye upon these drafted men and substitutes, but I think most of them will make good soldiers after they get tempered to the work.

Monday, August 24. I wrote home today on a sheet of note paper taken from a rebel soldier's knapsack at Falling Waters, where we crossed the Potomac July 14, when we were pursuing Lee after Gettysburg. I have carried it ever since, but did not think to use it until today. It is of a dirty brown color, coarsely ruled, and not very good in quality, yet it answers their purpose just as well, I presume, as though it were made in Belfast. Yesterday was the hottest day of the season, and a julep and an iced lemonade were refreshing. These are luxuries which we can now boast, as I get 150 pounds of ice a day. The consequence is I have plenty of company—visitors who call for a drink of ice-water.

August 28. We are having beautiful moonlight nights now, and every evening a cavalcade of eight or ten officers visits my quarters, when we all go out for a ride, generally taking the Bealton Road; after riding about four miles we all return to my tent, where an hour or two is spent in conversation, smoking, cobblers, etc. Two more deserters were shot in the 2d Division today. The entire Division was drawn up in line, and the doomed men marched to the place of execution behind their coffins, which were drawn in a wagon. The guard detailed for the shooting made bungling work, which added to the horrifying nature of the spectacle.

August 31. The month closes with quietude, and we are still at Morrisville; I think, however, that we shall not remain here much longer. Things military seem to be pointing to a movement of some kind.

September 1. I hear from home of a visit to my wife by Mrs. Bidwell, which was a pleasant reminder of the winter of 1861-2 at Camp Griffin. The 2d Corps has gone on a reconnoisance to Falmouth, leaving one regiment to guard the camp and the sick. so I am in supreme authority today. Quartermaster Snyder is quite sick in the hospital.

September 4. The Corps returned today, without having met with any special adventures. It seems the purpose of the movement was to aid in the destruction of some gunboats the enemy had placed in the Rappahannock; but I never heard whether the boats were even seen by any man of the command. Dr. Mary Walker rode up to the hospital just at dusk last evening, and I gave her quarters for the night. She appears to be one of those women cranks, who imagine they have a mission to perform in the Army, and so pester the doctors with their care.

September 5. These are quite busy days, as I have several very sick men with typhoid fever, dysentery, etc. I have lost but one patient since assuming charge of the 9th, out of over 150 different patients treated, and now have 65 on hand. I made application last Sunday, the 30th, for leave for seven days to go home to Mary Bostwick's wedding, but, as the Corps moved out on the reconnoisance to Falmouth on Monday and did not return till yesterday, it is doubtful if I get it in season to reach Lancaster by the 9th. The mails come with regularity now which is a great comfort, furnishing letters and papers from home almost daily.

Wednesday, September 9. The supply of ice and lemons is ample, and I am thus enabled to make the men in hospital very comfortable, during this very hot weather. This evening the wedding takes place at home, but I am not there as I hoped to be. This is the lot of a soldier—doomed to disappointment—but if life and health are only spared 'tis well.

Friday, September 11. My leave came last night and I started this morning, reaching Alexandria late in the afternoon, owing to the delay of the train. I engaged a livery man to drive me over to Washington in time for the six o'clock train north, for which he gets $5.00 if we succeed. On the Long Bridge we met a long train of army wagons which promised, at first, to impede our passage, but, as I happened to have a cavalry sabre with me that I

picked up on the field at Bealton after a cavalry fight, and which I was taking home, it was supposed that I was a staff officer with important despatches, as I waived a large envelope and motioned the train to give way to the left. This the officer in charge readily obeyed. The roadway across the bridge was cleared, and we reached the Baltimore & Ohio depot in season for the train.

I arrived home Saturday evening, the 12th, much to the surprise of everybody, not to say their gratification. On Monday I had a new plate of teeth made by Dr. Theo. G. Lewis, started back for the Army on Wednesday, and reached Washington on Thursday. I remained over Friday, the 18, in Washington to get my pay for August. Friday evening I attended Ford's Theatre, where I met Dr. and Mrs. S. B. Hunt and their son. I sat beside Mrs. Hunt during the play, and had a delightful visit with her. Dr. Hunt has medical charge of convalescent camp near Alexandria, and says he would make me executive officer if my rank was not so high. I could not think of advancing backwards like a crab, even with so tempting an offer as that; for I am well pleased with my present position, and think it is all I deserve at my age. I hear that the 2d Corps has moved out beyond Culpepper C. H., since my departure.

